# Two versus Three Infusion Regimens of N-Acetylcysteine for Acetaminophen Overdose

**DOI:** 10.3390/pediatric16010020

**Published:** 2024-03-20

**Authors:** Shadi Tamur, Bader Alyahya, Faisal Alsani, Ammar Abdulraheem Bahauddin, Maryam Aljaid, Sultan Al-Malki, Ahmad Alzahrani, Abdullah Khayat, Anwar Shams, Dominic S. Chalut

**Affiliations:** 1 Department of Pediatrics, College of Medicine, Taif University, P.O. Box 11099, Taif 21944, Saudi Arabia; maryam@tu.edu.sa (M.A.); sultanab@tu.edu.sa (S.A.-M.); as.alzahrani@tu.edu.sa (A.A.); khayatam@tu.edu.sa (A.K.); 2 Department of Emergency Medicine, College of Medicine, King Saud University, Riyadh 11451, Saudi Arabia; balyahya@ksu.edu.sa; 3 Department of Pediatrics, Jim Pattison Children’s Hospital, University of Saskatchewan, Saskatoon, SK S7N 5A2, Canada; faisal.alsani@usask.ca; 4 Department of Pharmacology and Toxicology, College of Pharmacy, Taibah University, Madinah 42361, Saudi Arabia; abahauddin@taibahu.edu.sa; 5 Department of Pharmacology, College of Medicine, Taif University, P.O. Box 11099, Taif 21944, Saudi Arabia; a.shams@tu.edu.sa; 6 Centre of Biomedical Sciences Research (CBSR), Deanship of Scientific Research, Taif University, Taif 21974, Saudi Arabia; 7 High Altitude Research Center, Taif University, P.O. Box 11099, Taif 21944, Saudi Arabia; 8 Department of Pediatrics and Epidemiology and Biostatistics, The Division of Emergency Medicine, The Montreal Children’s Hospital, McGill University Health Centre, Montreal, QC H4A 3J1, Canada; dominic.chalut@mcgill.ca

**Keywords:** two infusions of N-Acetylcysteine, pediatric acetaminophen toxicity, new N-Acetylcysteine regimen

## Abstract

Background: Acetaminophen overdose is a common clinical condition, often leading to liver toxicity. Current treatments involve the three-infusion N-Acetylcysteine (NAC) regimen (FDA-labeled), which may be complex, time-consuming, and need to be changed. An alternative uses two infusions instead, which offers possible advantages regarding simplicity and administration errors. This study sought to compare the respective efficacies and safety outcomes when treating acute acetaminophen overdose among children and adolescents. Methods: At Montreal Children’s Hospital, a retrospective study was conducted comparing pre-2003 FDA-labelled three-infusion NAC therapy with a two-infusion regimen. Information was collected regarding patient demographics, NAC administration details, errors, rates of hepatotoxicity, and adverse reactions, and the statistical test Chi-square test was employed to obtain the results. Results: A total of 126 patients met the inclusion criteria. Of these patients, 65 received a two-infusion regimen, and 61 patients received the FDA-labeled regimen. The two-infusion group experienced significantly fewer administration errors (4 errors vs. 23 errors; *p* < 0.001), while the rates of hepatotoxicity between them were similar. There were no instances of liver transplantation or mortality due to either regimen. Adverse reactions occurred equally frequently between both regimens with no discernible difference—the meantime to administer NAC was 9 h for the two-infusion regimen and 8.5 h for FDA-labeled regimen groups, respectively. Three cases of hepatitis were successfully treated with timely NAC therapy, and no liver transplantation or mortality occurred. Adverse reactions, including anaphylactoid reactions, were observed in both groups but were resolved when temporarily stopped and restarted at a slower infusion rate. Conclusions: The two-infusion NAC regimen proved similar efficacy at protecting liver damage and improving patient outcomes compared to its FDA-labeled three-stage counterpart, with significantly fewer administration errors for this version of NAC treatment, suggesting potential advantages in terms of safety and simplicity. Future research should investigate larger cohorts and more variables to validate these results further and optimize the management of acetaminophen overdose cases; further investigation should focus on dosing strategies, personalized approaches, and long-term patient care in this context.

## 1. Introduction

Acetaminophen is a widely used analgesic and antipyretic. It is the leading cause of acute liver failure in the United States [1,2]. Acetaminophen is generally safe when used at recommended doses, but its metabolism can produce a toxic metabolite called N-Acetyl-p-benzoquinone imine (NAPQI) [3,4]. Acetaminophen toxicity can lead to oxidative stress and liver damage, with potentially lethal outcomes [5]. Liver dysfunction and hepatocellular necrosis can result in acute liver failure, necessitating transplantation as the only option for survivors. Furthermore, complications such as renal failure, metabolic acidosis, coagulopathies, and encephalopathy may threaten patient wellbeing [6]. Acetaminophen toxicity is typically classified into four stages. Stage I, occurring within 24 h of an overdose, involves nonspecific symptoms like nausea and vomiting. Stage II, between 24 and 72 h, is characterized by right upper quadrant pain and liver tenderness, indicative of liver injury. Stage III, from 72 to 96 h, is crucial and characterized by the peak of hepatotoxicity, potentially leading to acute liver failure. Stage IV, beyond 96 h, involves recovery or progression to fatal liver failure. The impact on patient outcomes and healthcare resources makes effective prevention and management strategies even more vital [7].

Acetaminophen (APAP) overdose is a leading cause of liver toxicity and significant clinical concern [8,9]. To protect against liver damage after an APAP overdose, the prompt administration of N-Acetylcysteine (NAC), which is a glutathione analog, has long been recognized as the standard treatment [10]. Although the current FDA-labeled IV NAC regimen includes three doses over several hours, evidence suggests that two-infusion IV NAC regimens may provide equivalent protection [9,10].

### 1.1. Problem Context

The FDA-approved three-stage IV NAC regimen has become widely utilized and has shown promising results in protecting liver damage from an APAP overdose [11]. This recommendation is supported by studies demonstrating NAC’s efficacy in reducing morbidity and mortality associated with acetaminophen overdose [12]. However, this regimen can be complex and time-consuming and may not be appropriate for all patient populations, particularly children.

### 1.2. The Rationale for Two-Infusion IV NAC Regimen

Multiple studies suggest that a two-infusion IV NAC regimen may provide comparable hepatic protection during an APAP overdose, especially among children [9,10,13]. They demonstrate how an initial loading dose followed by one maintenance infusion achieves therapeutic plasma concentrations of NAC while replenishing glutathione levels [14]. This replenishment helps clear away toxic reactive metabolites formed during the metabolism of APAP metabolism [3].

Wong et al. conducted a comparative analysis between a three-stage IV NAC regimen and a two-infusion regime in patients who overdosed with APAP and found similar clinical outcomes between both regimens in terms of protecting liver damage and improving patient survival [9].

Syafira et al. also highlighted the potential advantages of an IV NAC regimen in preventing acute liver injury [10], with two infusions rather than long infusion periods potentially improving compliance and treatment adherence—this was supported by Wong et al., who reported improved patient compliance using such an approach in their research study [9].

The Montreal Children’s Toxicology Service adopted a two-infusion regimen in 2003. The change was driven by the observation of significant interruptions of NAC therapy between the second and third infusion, resulting in an increased rate of anaphylactoid reactions following the reintroduction of NAC.

This retrospective chart review compares the efficacy and safety outcomes of the FDA-labeled three-infusion N-Acetylcysteine (NAC) regimen with two infusion regimens in treating acute acetaminophen overdose among children and adolescents. Medical records will be utilized to track any development of acute hepatitis, acute liver failure, infusion errors, or adverse drug reactions during treatment.

## 2. Methodology

This retrospective chart review study assesses the effect of a protocol change by comparing a pre-2003 FDA-labeled three-infusion N-Acetylcysteine (NAC) regimen with a two-infusion regimen at the Montreal Children’s Hospital, Montreal, Canada. The FDA-labeled regimen consists of a loading dose of 150 mg/kg infused over 60 min, followed by a second dose of 50 mg/kg infused over 4 h (12.5 mg/kg/h), and finally, a third dose of 100 mg/kg infused over 16 h (6.25 mg/kg/h) [11]. The Montreal Children’s Hospital two-infusion regimen consists of a loading dose of 150 mg/kg over 50 min followed by a second infusion of 150 mg/kg over 20 h. The study received ethical approval from McGill University Scientific and Review Committees. In this study, patients aged 18 years or younger who received intravenous NAC for acute acetaminophen overdose were included. Exclusion criteria included individuals with chronic liver disease, those who were treated at another institution for acetaminophen overdose, those who received intravenous NAC for another indication, those taking oral NAC or with their NAC infusion terminated due to sub-toxic levels, those who had hepatitis, chronic acetaminophen toxicity or who took extended-release acetaminophen formulations.

For this study, acute hepatitis was defined as aspartate transaminase (AST) or alanine transaminase (ALT) levels exceeding 1000 U/L, while acute liver failure was defined as ALT or AST levels above 1000 U/L with an INR > 1.5 and hepatic encephalopathy. Deviations in the NAC dose by 10% or more from either the FDA-labeled or the new two-infusion protocol were considered an incorrect dose. In comparison, an incorrect rate was characterized by an NAC bolus administration in less than 15 min or a deviation of 10% or more from the respective protocol infusion rate. The interruption of therapy entailed any treatment halt lasting one hour or more.

### 2.1. Data Collection

Data collection involved searching the medical record database using ED discharge diagnoses related to “poisoning/overdose/intoxication, acetaminophen” to identify cases of acetaminophen overdose. A data collection sheet was employed to gather information including patient age, sex, type and the formulation of acetaminophen product, the dose of acetaminophen ingested, total NAC dose, duration of infusion, adverse reactions to NAC, time from an overdose to initiation of NAC therapy, length of stay, type of acetaminophen overdose (intentional or accidental), prescription errors, time to acetaminophen concentration measurement, and laboratory values. Data were collected by a single individual (ST) to minimize errors.

### 2.2. Data Analysis

Data analysis involved reporting continuous variables as means and nominal data as frequency distributions. The rates of hepatotoxicity and administration error frequencies were compared between groups using the chi-square test with SPSS version 11.0.1 for Windows (SPSS, Inc., Chicago, IL, USA).

## 3. Results

**Figure 1:**
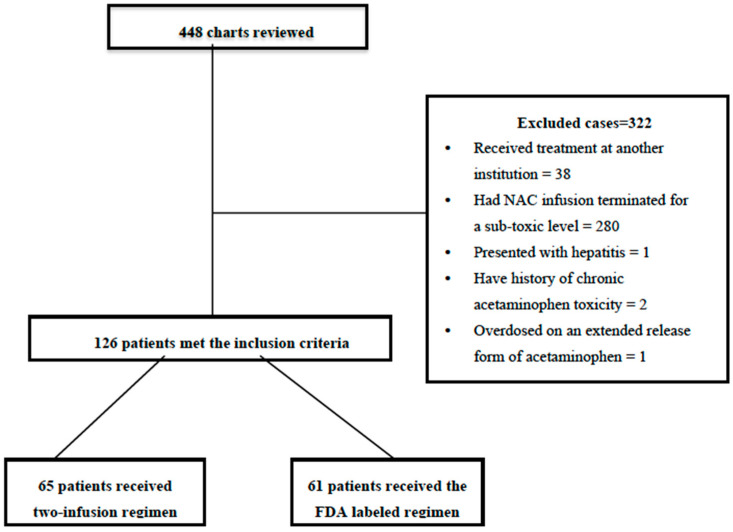
Study flow diagram.

As part of this study period, 448 patients received intravenous N-Acetylcysteine (NAC) for acute acetaminophen overdose (Figure 1). After exclusion criteria were fulfilled for each (Table 1), 126 individuals met the inclusion criteria and were included for analysis (Figure 1). Table 1 presents the baseline demographic information about each included patient as follows: age, gender, mean time to NAC administration, and proportion of intentional ingestions, among others (Figure 1). Table 1 presents baseline demographic details about each included patient, including age, gender, and mean time to NAC administration, as well as intentional ingestion rates by age and gender for each, co-ingestions, and comorbidities.

Table 1 presents the baseline demographics of patients receiving intravenous N-Acetylcysteine (NAC) to treat acute acetaminophen overdose. Average patient ages were similar between the two-infusion regimen and FDA-labeled regimen groups, at 14.4 years and 14.6 years, respectively. Female patients dominated both groups, accounting for 86% in the two-stage regimen group and 90% in the FDA-labeled regimen group. The mean times to administer NAC were comparable between groups, with 9 h for the two-infusion regimen and 8.5 for FDA-labeled regimen groups, respectively, from the time of ingestion. Furthermore, most ingestion in both groups was intentional: 58% in the two-infusion regimen group and 62% in the FDA-labeled regimen group, respectively.

Table 2 summarizes a comparison between the two groups concerning the dose ingested in mg/kg and initial acetaminophen concentrations; the dose-ingested mg/kg are similar between the two groups. For the two-infusion regimen, there were six patients with an unknown amount ingested. For the FDA-labeled regimen, there were four patients with unknown ingestion. There was no statistically significant difference between both groups when we compared the acetaminophen concentration; since there were patients who presented after 8 h post-ingestion, we subdivided the two groups into concentrations obtained 4–8 h post ingestions and concentrations obtained 8–24 h post-ingestion. In the two-infusion group, 23 patients had levels obtained 8–24 h post-ingestion. In the FDA-labeled group, 13 patients had levels obtained 8–24 h post-ingestion. We compared the errors identified within both the two-infusion regimen and FDA-labeled regimen groups. Errors observed within the two-infusion regimen group included two administration errors, one incorrect dose, and one missing loading dose. No infusion stopped for less than 21 h, and no interruptions to therapy lasting over one hour were noted. Conversely, the FDA-labeled regimen group experienced six administration errors: one incorrect dose and one missed loading dose were noted, as were one instance where infusion stopped for less than 21 h and 15 interruptions to therapy lasting over 1 h. Comparing both regimens revealed significantly greater error rates for the FDA-labeled regimen versus the two-infusion regimen (23 errors vs. 4, respectively, with *p*-value = 0.001). These findings indicate a higher rate of errors among participants using the FDA-labeled regimen compared with the two-infusion regimen, suggesting the latter had significantly less chance of error when administrating N-Acetylcysteine (NAC) for acute acetaminophen overdose.

Table 3 details cases of hepatitis among the patients. It provides details for three individuals who experienced hepatotoxicity, such as their age and gender, as well as the dosage ingested per kg of body weight in milligrams per kilograms (mg/kg), the peak AST or ALT levels in U/L units, and milligrams per deciliters of APAP per milliliter deciliter per hour of administration time to the NAC administration protocol received, peak INR levels, adverse reactions experienced and outcomes.

Notably, no significant differences were observed in rates of hepatotoxicity between the two regimens. All patients recovered without sequelae from either regimen, with no instances of liver transplantation or mortality recorded. One patient using the FDA-labeled regimen did have an elevated peak INR (2.77), although transaminase levels did not elevate as expected from NAC therapy. Table 3 illustrates this point further by showing all three cases of hepatitis receiving timely NAC therapy—whether via a two-infusion regimen or FDA-labeled regimen—with successful treatments employed both ways. Adverse reactions occurred in two instances and recovered without needing transplantation. Regardless, adverse events did not require transplantation.

The results show that among all the included patients, there were significantly more administration errors with the FDA-labeled regimen compared to the two-infusion protocol (23 errors against four errors, respectively, *p*-value 0.001). Most frequently seen were interruptions of infusion for more than 1 h. Adverse reactions, including anaphylactoid reactions, occurred in both groups, with three patients experiencing this reaction in the two-infusion regimen and four in the FDA-labeled regimen, respectively—typically during infusion but resolved when temporarily stopped and restarted at a slower infusion rate.

## 4. Discussion

This study’s results are along the lines of the results of several similar studies indicating that the two-infusion regimen is as effective as the FDA-labeled three-stage regimen for administering N-Acetylcysteine (NAC) to children and adolescents experiencing acute acetaminophen overdose. The results demonstrated that both protocols offer similar hepatic protective properties, evidenced by comparable rates of hepatotoxicity and favorable patient outcomes. The two-infusion regimen also had significantly fewer administration errors, suggesting potential benefits over its counterpart.

The demographic characteristics of the patients receiving intravenous N-Acetylcysteine (NAC) to treat acute acetaminophen overdose are presented in Table 1.

Most of our patients were female, which is explained by the fact that the rate of suicidal attempts is much higher in this age and gender. A systematic review and meta-analysis looking at gender differences in suicidal behavior in adolescents and young adults found females were more likely to attempt suicide (two-fold higher risk than males) while males were more likely to die by suicide [15].

In our cohort, only four patients were under the age of 12 years, with this finding likely being because toxic ingestion is more common with intentional ingestion compared to accidental ingestion, which is more common in young children; NAC treatment was terminated in 280 patients due to subtoxic ingestion, and these patients were excluded.

Wong and Graudins noted similar average ages among patients enrolled in two- and three-bag regimen groups (14.4 years on average, respectively) [16]. Schmidt et al. (2018) did not provide specific age data but noted similar demographic characteristics between both regimens [17]. Regarding gender distribution, Wong and Graudins (2016) observed that most of the patients in both two-infusion and three-infusion regimen groups were female (86% and 90%, respectively) [16]. Schmidt et al. (2018) found that both regimens had similar demographic characteristics between them [17].

Similarly, we compared two regimens in terms of the mean time to administer NAC. Wong and Graudins (2016) reported an approximate mean administration time of 9 h using a two-infusion regimen [16], while McNulty et al. (2018) reported 10 h as their median NAC administration duration for the single-infusion regimen. Schmidt et al. (2018) did not provide specific timing details but noted that their modified two-infusion protocol was introduced in 2015 [17]. Wong and Graudins reported intentional ingestion rates in 58% of cases for two-infusion regimen groups and 62% of cases in three-infusion regimen groups [16]. No data were provided by Schmidt et al. regarding intentional ingestion [17]. The findings from these studies indicate that the baseline demographics of patients receiving NAC for an overdose were similar in the two-infusion and FDA-label regimen groups, including their average age, gender distribution, and percentage of intentional ingestions across studies.

In our cohort, there were 29 patients in the FDA-labeled regimen, and 24 patients in the two-infusion regimen received Activated Charcoal (AC). AC administration usually depends on the time between ingestion and presentation and ideally should be administered within one-hour post-ingestion. Most adolescent patients are late presenters post toxic ingestion; in our cohort, the mean presentation time to the emergency department was 8 h, which led to less patient-received-activated charcoal, and the mean initiation time of NAC from the time of ingestion was 8.5 h for the two-infusion regimen and 9 h for the FDA-labeled regimen group, respectively. In our hospital practice, we start empirical NAC if the calculated dose mg/kg is at the toxic level of 150 mg/kg until the APAP level is obtained; if there is a sub-toxic level, we discontinue NAC. In fact, the above-mentioned initiation time is not because we do not use oral preparation or we delay the NAC administration pending the APAP result; it is mainly due to the later presentation of the patient themselves.

Our findings on total errors and descriptions of all errors gave us critical insights into administering N-Acetylcysteine (NAC) for treating acetaminophen overdose. They showed a significantly higher error rate in the FDA-labeled regimen versus the two-stage regimen (23 errors vs. 4 errors; *p*-value: 0.001).

Bateman et al.’s research underscores the need for improved regimens to minimize adverse events and errors associated with NAC administration [18]. Their two-infusion regimen with a slower initial infusion rate demonstrated lower rates of adverse events compared with the original 20.25 h regime—similar results can be seen here, with significantly fewer errors committed with our two-infusion regimen.

Yamamoto et al. and Bailey et al. present complementary findings regarding adverse drug reactions (ADRs) associated with NAC treatment and, specifically, those associated with Acetaminophen overdose patients [19,20]. Their studies highlight the incidence and management of ADRs due to NAC use. Our results, showing a reduced rate of errors during two-stage regimen administration, suggest that such factors could help minimize errors or interruptions that lead to ADRs.

Bailey et al. examined dosing variations for Acetaminophen overdose treatment using infusions of acetylcysteine [20]. Their research indicated some variability in administered concentrations, which could increase the risk of treatment failure or adverse effects. Although not directly addressed in our results, standardizing the concentration of acetylcysteine infusions could potentially reduce dosing errors and enhance treatment outcomes.

Chiew et al’s. research explores the current three-infusion intravenous regimen for Acetylcysteine administration and highlights the modifications needed to decrease adverse reactions [21], specifically by slowing the loading dose rate. They note that modified regimens aiming to lower adverse event rates by slowing loading dose rates have shown promise. Though our results compared two-infusion to FDA-labeled regimens, Chiew’s insights align with our goal of minimizing errors and adverse events during its administration.

The results from these three cases of hepatitis offer invaluable insights into the treatment outcomes for patients who received timely N-Acetylcysteine (NAC) therapy using either the two-infusion or FDA-labeled regimens. It is essential to compare and contrast these results with other research to comprehend their implication better. In our study, all three patients received NAC therapy within the recommended timeline for treating an acetaminophen overdose. All three experienced various levels of hepatotoxicity as indicated by peak AST or ALT levels, APAP levels, and INR levels. Nevertheless, all recovered without needing liver transplantation or death from such severity of effects.

Comparing our findings with other studies, Marks et al. conducted a retrospective observational study on outcomes following massive Acetaminophen overdose [22], reporting on a larger cohort and finding higher risks of organ damage even when NAC was administered early. Our smaller sample study offered similar successful treatment outcomes and the resolution of hepatitis without transplantation, as Marks et al. reported [22].

Fathelrahman conducted an insightful research study that provided solutions for managing Acetaminophen overdose, including high-risk patients, late presentations, and co-administered medications [23]. While our case results did not directly focus on these specific scenarios, their positive outcomes suggest an important lesson about managing acetaminophen overdose: timely NAC therapy administration plays a vital role in protecting liver tissue damage while expediting recovery.

In our study, two patients experienced anaphylactoid reactions during NAC therapy. This observation corresponds to Yamamoto et al., who examined the incidence and management of NAC-related anaphylactoid reactions [19]. Such reactions can arise during its administration and should be managed accordingly by temporarily stopping its infusion if necessary. Regardless, patients in our study recovered without long-term consequences due to these adverse events.

While the absence of specific details regarding APAP exposure (such as APAP levels and doses) might be considered a gap in knowledge, the study still presents a valid case for the efficacy of the two-infusion NAC regimen. It did show a significant reduction in administration errors compared to the FDA-labeled three-infusion regimen. Additionally, this study demonstrated comparable hepatic protection between the regimens, emphasizing the importance of timely NAC administration.

Overall, while adding APAP exposure details (APAP level and dose) would have strengthened the comparison further, this study still provides a valuable understanding of the advantages of the two-infusion NAC regimens in treating acetaminophen overdose among pediatric populations.

Overall, our three cases of hepatitis, along with comparing and contrasting their findings to other study findings, demonstrate the value of early NAC therapy in successfully treating acetaminophen overdose. Both the two-infusion NAC and the FDA-labeled regimens proved efficacious at treating the overdose and avoiding liver transplantation. Adverse reactions such as anaphylactoid reactions should be closely monitored and managed accordingly. Further research should validate these findings among larger cohorts and explore strategies to enhance the management of complications related to overdose reactions caused by overdose complications.

## 5. Limitations

Sample size: As with any research study, this one has its share of limitations that should be acknowledged. This study was conducted with a limited sample size of 342 cases. There is a need for a larger sample size to confirm the differences we observed in the administration error rates.

Patient population: This study focused on a specific patient population. This limits the generalization of the findings to other populations with different characteristics, such as age, comorbidities, and severity of overdose.

Short-term outcomes: this study only assessed short-term outcomes (resolution of hepatitis). Investigating the long-term effect of three and two intravenous regimens on patient’s health is crucial.

Additional variables not considered: This study should consider more applicable variables that could influence treatments and patient’s health. For example, the time from overdose to treatment initiation, the existence of other medications, and individual differences in metabolism.

Missing information on dosing strategies: the conclusion mentions the need for further research on optimal dosing strategies, but it does not provide any specific suggestions or hypotheses about it.

Overall, this paper provides valuable insights into the potential benefits of the two-infusion NAC regimen for acetaminophen overdose. However, further research with a larger sample size, a broader population, and a more extended follow-up period is needed to confirm the findings and optimize treatment strategies for this potentially life-threatening condition.

## 6. Future Research Implications

Several areas of future research can be suggested to address these limitations. First and foremost, larger-scale studies involving diverse patient populations must be conducted to increase generalizability. It would also allow for a more comprehensive evaluation of different regimens and their efficacy across patient populations. Examining the long-term outcomes and follow-up of patients experiencing hepatotoxicity would provide valuable insights into its possible long-term consequences and any necessary interventions or monitoring plans. Optimizing NAC dosing regimens to minimize adverse reactions while meeting therapeutic efficacy requirements could further enhance outcomes and help minimize errors associated with administering this therapy. Furthermore, exploring alternative routes of administration or tailored dosing strategies based on individual patient characteristics could further optimize the results while preventing errors associated with its administration. Ongoing research efforts must focus on increasing our understanding of acetaminophen overdose management by considering various factors that might impede treatment outcomes and optimizing therapeutic strategies in pursuit of the best patient outcomes.

## 7. Conclusions

This study investigated two N-Acetylcysteine (NAC) administration regimens in cases of an overdose of acetaminophen. Both regimens involved two steps of NAC administration for optimal results. The findings revealed a significantly lower rate of administration errors with the two-stage regimen compared to the FDA-labeled regimen. This highlights the significance of adopting modified regimens to minimize errors and ensure patient safety when treating an acetaminophen overdose. Furthermore, this study revealed that both regimens were equally successful at treating hepatotoxicity with favorable outcomes and the resolution of hepatitis in all cases. As this research focused on only a limited sample size and patient population, more comprehensive research using larger cohorts with additional variables should be conducted to maximize the care of those taking an overdose of acetaminophen. Future studies should examine optimal dosing strategies, tailored approaches, and long-term outcomes to improve the management of an acetaminophen overdose and provide the highest standard of patient care possible.

## Figures and Tables

**Table 1:** Patient’s demographics and description of the ingestions.

	Two-Infusion Regimen	FDA-Labeled Regimen
Age (year)	14.4	14.6
Gender (F)	(56) 86% 212 (229, 351) 9	(55) 90% 240 (258, 411) 8.5
Mean time to NAC (hour)		
Intentional ingestion	58 (%)	62 (%)
Co-ingestions (number of patients)	Ibuprofen (5) Iron (1) Iron, Vitamin D (1) Benzodiazepine, Litium (1) Paroxetine, Quitapine (1) Naproxen (2) Aspirin, Dimenhydranate (1) Aspirin, Caffeine (1) Aspirin, Enalopril, Metoprolol (1) Caffeine (1) Codeine, caffeine (1) Paroxetine (1)	Ibuprofen (4) Iron (1) Lithium, Thyroxine, Olanzepine, Venlafaxine (1) Naproxen (1) Aspirin (3) Aspirin, Dimenhydranate (1) Nail polish removal, Paroxetine, Codeine (1) Phenytoin and phenobarbital (1) Alcohol overdose (2) Paroxetine (1)
Past medical histroy (number of patients)	Depression (10) Asthma, depression (2) Migraine, depression (1) Asthma (2) Bipolar disorder (1) Anorexia nervosa (3) Anorexia nervosa, depression (2) Bulemia nervosa (1) ADHD (2) Iron defecincy anemia (1) Migraine (2) Panic attack (2)	Depression (6) Depression, pregnancy (1) Migraine, depression (1) Asthma (1) Bipolar disorder (1) Anorexia nervosa (1) Anorexia nervosa, depression (1) Bulemia nervosa, depression (1) Migraine (2) Iron defecincy anemia (1)

**Table 2:** Total number of errors and descriptions of all errors.

	Two-Infusion Regimen	FDA-Labeled Regimen	*p*-Value
Dose ingested mg/kg			
Median (IQR)	212 (229, 351)	240 (258, 411)	0.36
Acetaminophen concentration			
4–8 h post-ingestion	1060 (1054, 1398)	1155 (1086, 1402)	0.87
8–24 h post-ingestion	855 (669, 1153)		
Median (IQR)		459 (412, 836)	0.104
Errors			
Total (Number)	4	24	<0.001
Descriptions:			
Administration errors	2	6	
Incorrect dose	1	1	
Missing loading dose	1	1	
Infusion stopped < 21 h	0	1	
Interruption > 1 h	0	15	

**Table 3:** Cases of hepatitis.

	Case 1	Case 2	Case 3
Patients details			
Age (Y), gender	14 male	15 female	17 female
Dose ingested mg/kg	500	123	Unknown
Peak AST or ALT U/L	11,179	6874	2909
APAP level µmol/L	371 at 15 h	549 at 22.5h	471 at 11.5 h
Co-ingestion	Cannabinoid	-	-
Time to NAC (h:m)	22:50	15:02	15:05
Peak INR	4	2.55	1.63
Protocol	Two stage	Two stage	FDA
Adverse reactions			Anaphlactoid reaction Dose was held for >1 h
Outcome			
Hepatitis resolved, no transplant	Yes	Yes	Yes

## Data Availability

No new data were created or analyzed in this study. Data sharing is not applicable to this article.

## References

[1] Bauerlein D.K., Williams A.P., John P.R. (2021). Optimizing Acetaminophen Use in Patients with Risk Factors for Hepatotoxicity: Reviewing Dosing Recommendations in Adults. Pain Med..

[2] Budnitz D.S., Lovegrove M.C., Crosby A.E. (2011). Emergency department visits for overdoses of acetaminophen-containing products. Am. J. Prev. Med..

[3] Guengerich F.P. (2020). Cytochrome P450 2E1 and its roles in disease. Chem.-Biol. Interact..

[4] Chowdhury A., Nabila J., Temitope I.A., Wang S. (2020). Current etiological comprehension and therapeutic targets of acetaminophen-induced hepatotoxicity. Pharmacol. Res..

[5] Shekunov J., Lewis C.P., Voort J.L.V., Bostwick J.M., Romanowicz M. (2021). Clinical Characteristics, Outcomes, Disposition, and Acute Care of Children and Adolescents Treated for Acetaminophen Toxicity. Psychiatr. Serv..

[6] Shalimar, Acharya S.K., Kumar R., Bharath G., Rout G., Gunjan D., Nayak B. (2020). Acute Liver Failure of Non-A-E Viral Hepatitis Etiology-Profile, Prognosis, and Predictors of Outcome. J. Clin. Exp. Hepatol..

[7] Yoon E., Babar A., Choudhary M., Kutner M., Pyrsopoulos N. (2016). Acetaminophen-Induced Hepatotoxicity: A Comprehensive Update. J. Clin. Transl. Hepatol..

[8] Sudanagunta S., Camarena-Michel A., Pennington S., Leonard J., Hoyte C., Wang G.S. (2023). Comparison of Two-Bag Versus Three-Bag N-Acetylcysteine Regimens for Pediatric Acetaminophen Toxicity. Ann. Pharmacother..

[9] Wong A., Isbister G., McNulty R., Isoardi K., Harris K., Chiew A., Greene S., Gunja N., Buckley N., Page C. (2020). Efficacy of a two bag acetylcysteine regimen to treat paracetamol overdose (2NAC study). EClinicalMedicine.

[10] Syafira N., Graudins A., Yarema M., Wong A. (2022). Comparing development of liver injury using the two versus three bag acetylcysteine regimen despite early treatment in paracetamol overdose. Clin. Toxicol..

[11] Pauley K.A., Sandritter T.L., Lowry J.A., Algren D.A. (2015). Evaluation of an Alternative Intravenous N-Acetylcysteine Regimen in Pediatric Patients. J. Pediatr. Pharmacol. Ther..

[12] Prescott L., Ballantyne A., Proudfoot A., Park J., Adriaenssens P. (1977). Treatment of paracetamol (acetaminophen) poisoning with N-acetylcysteine. Lancet.

[13] Bateman D.N., Dear J.W., Thanacoody H.K.R., Thomas S.H.L., Eddleston M., A Sandilands E., Coyle J., Cooper J.G., Rodriguez A., Butcher I. (2014). Reduction of adverse effects from intravenous acetylcysteine treatment for paracetamol poisoning: A randomised controlled trial. Lancet.

[14] Ye H., Nelson L.J., del Moral M.G., Martínez-Naves E., Cubero F.J. (2018). Dissecting the molecular pathophysiology of drug-induced liver injury. World J. Gastroenterol..

[15] Miranda-Mendizabal A., Castellví P., Parés-Badell O., Alayo I., Almenara J., Alonso I., Blasco M.J., Cebrià A., Gabilondo A., Gili M. (2019). Gender differences in suicidal behavior in adolescents and young adults: Systematic review and meta-analysis of longitudinal studies. Int. J. Public Health.

[16] Wong A., Graudins A. (2016). Simplification of the standard three-bag intravenous acetylcysteine regimen for paracetamol poisoning results in a lower incidence of adverse drug reactions. Clin. Toxicol..

[17] Schmidt L.E., Dalhoff K., Poulsen H.E. (2002). Acute versus chronic alcohol consumption in acetaminophen-induced hepatotoxicity. Hepatology.

[18] Bateman D.N., Dear J.W., Thomas S.H. (2016). New regimens for intravenous acetylcysteine, where are we now?. Clin. Toxicol..

[19] Yamamoto T., Spencer T., Dargan P.I., Wood D.M. (2014). Incidence and management of N-acetylcysteine-related anaphylactoid reactions during the management of acute paracetamol overdose. Eur. J. Emerg. Med..

[20] Bailey G.P., Wood D.M., Archer J.R.H., Rab E., Flanagan R.J., Dargan P.I. (2017). An assessment of the variation in the concentration of acetylcysteine in infusions for the treatment of paracetamol overdose. Br. J. Clin. Pharmacol..

[21] Chiew A.L., Isbister G.K., Duffull S.B., Buckley N.A. (2016). Evidence for the changing regimens of acetylcysteine. Br. J. Clin. Pharmacol..

[22] Marks D.J.B., Dargan P., Archer J.R.H., Davies C.L., Dines A.M., Wood D., Greene S.L. (2017). Outcomes from massive paracetamol overdose: A retrospective observational study. Br. J. Clin. Pharmacol..

[23] FAthelrAhmAn A.I. (2021). Ten challenges associated with management of paracetamol overdose: An update on current practice and relevant evidence from epidemiological and clinical studies. J. Clin. Diagnost Res..

